# Real-Time Context-Aware Recommendation System for Tourism

**DOI:** 10.3390/s23073679

**Published:** 2023-04-02

**Authors:** JunHo Yoon, Chang Choi

**Affiliations:** Department of Computer Engineering, Gachon University, 1342 Seongnam-daero, Sujeong-gu, Seongnam-si 13120, Gyeonggi-do, Republic of Korea; junho6257@gachon.ac.kr

**Keywords:** real-time context-aware, recommendation system, tourism, AI

## Abstract

Recently, the tourism trend has been shifting towards the Tourism 2.0 paradigm due to increased travel experiences and the increase in acquiring and sharing information through the Internet. The Tourism 2.0 paradigm requires developing intelligent tourism service tools for positive effects such as time savings and marketing utilization. Existing tourism service tools recommend tourist destinations based on the relationship between tourists and tourist destinations or tourism patterns, so it is difficult to make recommendations in situations where information is insufficient or changes in real time. In this paper, we propose a real-time recommendation system for tourism (R2Tour) that responds to changing situations in real time, such as external factors and distance information, and recommends customized tourist destinations according to the type of tourist. R2Tour trains a machine learning model with situational information such as temperature and precipitation and tourist profiles such as gender and age to recommend the top five nearby tourist destinations. To verify the recommendation performance of R2Tour, six machine learning models, including K-NN and SVM, and information on tourist attractions in Jeju Island were used. As a result of the experiment, R2Tour was verified with accuracy of 77.3%, micro-F1 0.773, and macro-F1 0.415. Since R2Tour trains tourism patterns based on situational information, it is possible to recommend new tourist destinations and respond to changing situations in real time. In the future, R2Tour can be installed in vehicles to recommend nearby tourist destinations or expanded to tasks in the tourism industry, such as a smart target advertising system.

## 1. Introduction

Recently, there has been a change in the tourism trend towards the Tourism 2.0 paradigm due to the increase in travel experience worldwide and the spread of information acquisition and sharing through the Internet. The Tourism 2.0 paradigm seeks to create new value for the tourism industry by valuing the vitalization of information and communication and participatory cultural experiences, rather than simple travel and consumption activities [1]. In addition, Tourism 2.0 aims to give new value to the new tourism industry by developing information and technology, providing various experiences and cultures to tourists, and promoting the development of the local economy and environment by pursuing sustainable tourism. The free/foreign independent tour (FIT) is one of the representative tours of the Tourism 2.0 paradigm, and the demand for non-face-to-face services is increasing due to COVID-19 [2]. Due to FIT, tourists search, reserve and pay, and share information through online travel platforms and mobile applications, and the influence of social media and online platforms on the overall tourism industry is increasing [3]. FIT is required to develop intelligent tourism service tools with positive effects such as an improved understanding of tourist destinations and revisit rates, the development of tourism information technology, and improved marketing efficiency [4]. Travel planning and guidebook applications and tourism recommendation services, which are tourism service tools for FIT in the Tourism 2.0 paradigm, have advantages such as improved customer experiences, time and cost savings, data analysis, and marketing utilization [5]. For tourism service tools, research is being conducted on a system that recommends tourist destinations according to the relevance of tourists or tourist destinations [6]. Collaborative filtering (CF)-based recommendation, which recommends tourist destinations according to tourist relevance, is a method of finding similarities between users using information between users and items [7]. Content filtering (CB)-based recommendation, which recommends tourist destinations according to their relevance, is a method for determining the similarity between items using item information [8]. Handling new data without information is difficult for filtering-based tourist destination recommendation systems (RS) because of the required data size and cold start problem. Therefore, research on an RS using artificial intelligence (AI) is being conducted [9]. An AI-based RS is recognized as a key element capable of targeting marketing and personalized recommendations by providing appropriate information or products that users may prefer by subdividing them by user inclination and type [10].

Tourism service tools include a CF-based RS method that recommends tourist attractions according to their relevance to tourists and a CB-based RS method that recommends tourist attractions according to their relevance to tourist destinations [11]. The CF-based RS assumes that similar tourists have similar preferences for a specific tourist destination based on interaction data between tourists and tourist destinations [12]. Because CF is performed based on interactions between tourists and tourist destinations, recommendations can be made even if the similarity between tourist destinations is not high. However, new tourist destinations cannot be applied because no tourist information is available [13]. To solve the cold start problem, a CB-based RS that recommends tourist destinations according to their relevance can also recommend similar tourist destinations using tourist destination information [14]. CB can provide recommendations without interaction data between tourists and tourist destinations. However, its performance deteriorates compared with CF-based RS when sufficient data are collected [15]. An RS using AI can dynamically utilize the data size or characteristics because they use tourism patterns rather than the similarity between items, and they have achieved remarkable recognition performance with the development of algorithms and computation power [16]. However, RS based on tourism patterns using AI have limitations in dynamic situations because they do not reflect real-time changes in external factors and distance information, such as temperature or precipitation [17].

In this study, we proposed a real-time recommendation system for tourism (R2Tour) to respond to dynamic situations by reflecting real-time changing external factors and distance information and recommend customized tours according to the type of tourist. R2Tour recommends the top five nearby tourist destinations by learning a real-time context that includes real-time situational information and a tourist profile that includes the type of tourist using a machine learning (ML) model. The real-time context includes weather information, such as temperature and precipitation, and sequential information, such as season and location. The tourist profile includes gender, age, companion, and tour type. In this study, electric vehicle information (EVGPS) provided by the Korea Electric Power Corporation Knowledge Data Network (KEPCO KDN), the Visit Korea data lab, and Korea Meteorological Administration data were used to evaluate R2Tour. The results of evaluating R2Tour using information on tourist attractions, situations, and tourist profiles on Jeju Island, created by combining EVGPS, Visit Korea Data Lab, and Korea Meteorological Administration data, were verified with accuracy of 77.3%, micro-F1 0.773, and macro-F1 0.415. Our updated code, dataset, and trained models are available at https://github.com/junhoy00n/Real-time-Recommendation-for-Tourism (accessed on 3 March 2023).

The objectives and contributions of this thesis are summarized as follows.

- **Development of a customized travel destination RS according to real-time context changes**: It is common for existing tourism service tools to recommend destinations based on the relationship between tourists and tourist attractions or tourism patterns. However, recommendations are complex when there is insufficient information or situations that change in real time. In this paper, we try to overcome these limitations by proposing a real-time RS that recommends customized travel destinations according to external factors, distance information, and types of tourists.
- **Travel pattern analysis and prediction using ML models**: In this study, an ML model is trained using traveler profiles and contextual information to analyze and predict travel patterns. Through this, we can understand travelers’ preferences and behavior patterns and contribute to implementing a personalized RS.
- **Data collection and analysis for smart advertising system development**: In this study, the important process is to collect and analyze the data necessary to implement the RS. Through this, it is possible to understand the traveler’s preferences and behavioral patterns and derive useful information that can be utilized in the tourism industry, such as smart advertising systems.

The remainder of this paper is organized as follows. The related work is mentioned in Section 2 and consists of a filtering-based RS, ML model, and AI-based RS. The architecture of R2Tour is presented in Section 3. The experiment is mentioned in Section 4; the description of the data and environment used in the experiment and the experimental results are discussed; finally, the conclusions and future work are addressed in Section 5.

## 2. Related Work

Tourism service tools include a CF method that recommends tourist destinations according to the relevance to tourists and a CB method that recommends tourist destinations according to the relevance of tourist destinations. As it is difficult to handle the size of the required data and the cold start problem, research is being conducted to recommend tourist destinations based on tourism patterns using AI. The AI-based RS ignores the similarity between items and recommends tourist destinations based on tourism patterns. It has achieved excellent recognition performance with the advancement of algorithms and computation power. AI-based RS are recognized as critical elements capable of targeting marketing and personalized recommendations by providing appropriate information or products that users may prefer by subdividing them according to the user’s inclinations and types. The following section describes the AI model and AI-based RS presented in Table 1, and the filtering-based RS.

### 2.1. Filtering-Based Recommendation System

Tourism service tools include a CF method that recommends tourist destinations according to the relevance to tourists and a CB method that recommends tourist destinations according to the relevance of tourist destinations. The filtering-based RS analyzes the data input by tourists; learns their preferences, interests, and history of visits; and recommends travel destinations highly likely to be preferred [24]. Generally, a filtering-based RS collects and learns user profile information such as visited tourist attractions and travel styles. Hence, the RS matches tourist profiles and travel destination information to recommend new travel destinations. In other words, the filtering-based tourist destination RS is a system that filters and provides recommended tourist destinations according to the criteria preferred by tourists [25]. The CF-based RS is a method of recommending new tourist destinations by analyzing the similarity between tourists based on the evaluation information of the tourist destinations evaluated in the past by tourists. A CF-based RS can consider the preferences of tourists because it uses the information of a large number of tourists. In addition, when new tourist information is added, it is possible to recommend travel destinations preferred by other tourists with similar preferences to the current tourist. Moreover, since CF is performed based on interactions between tourists and tourist destinations, recommendations can be made even if the similarity between tourist destinations is not high. However, if the data evaluated by tourists are small or if the past evaluation information is different from the current preference, the recommendation result may not be accurate. Nonetheless, new tourist destinations cannot be applied because there is no tourist information. On the other hand, the CB-based RS recommends tourist attractions with characteristics similar to those reserved by tourists. The CB-based RS can provide recommendation results considering the tourist’s personal preferences. The recommendation can be made even if the data that the tourist evaluates are minimal. However, the recommendation result may be limited when the tourist’s preference information changes or the information input by the tourist is insufficient. In addition, since the CF-based RS utilizes the evaluation information of a large number of tourists, it is difficult to obtain appropriate recommendation results when the data are less dispersed. The CB-based RS utilizes the characteristic information of tourist destinations, so it is suitable for calculating the similarity between travel destinations. However, the recommendation results may be distorted if a statement that has not been used previously is used. Therefore, studying an RS using AI that can dynamically utilize the data size or characteristics is necessary because it uses tourism patterns rather than similarities between items [26].

### 2.2. Machine Learning Used for Recommendation System

ML is a technique for developing algorithms and statistical models that computer systems use to perform tasks without explicit instructions by relying on patterns and reasoning. Because ML focuses on predicting the behavior of a specific target based on learning from data using algorithms such as K-NN and support vector machine (SVM), it has been widely used in RS for particular information [27]. K-NN is an ML algorithm that assumes that data with similar characteristics belong to similar categories, and it predicts labels based on the K-nearest neighbors [28]. SVM defines a decision boundary or reference line and predicts labels based on the decision boundary [29]. Random forest creates a decision tree by randomly selecting only some features and predicting the label based on the most labels or the average value based on several decision trees produced by repeating it [30]. Voting is an ensemble ML algorithm that predicts the most significant number of labels based on multiple classifiers [31]. XGBoost combines several level-wise decision trees and learns a robust prediction model by applying a weight to the learning error and sequentially reflecting it in the next learning model [32]. Similar to XGBoost, LightGBM uses a combination of decision trees. In contrast to XGBoost, LightGBM uses a leafwise method to create decision trees [33]. ML algorithms such as K-NN and SVM enable computer systems to process historical data and identify their patterns. This enables us to predict the outcomes more accurately using a given input dataset [34].

### 2.3. AI-Based Recommendation System

RS is an information filtering technology that helps users to find the required information. With the increase in information, the importance of RS is increasing, and the technology of RS is advancing with the development of AI-based technology. Anwar et al. [18] proposed a K-NN-based RS using the user–item matrix of a Movie Trust dataset and verified it with an RMSE of 0.822. Elbir et al. [19] proposed an SVM-based RS using the acoustic features of the GTZAN dataset and verified it with accuracy of 97.6%. Guo et al. [20] proposed a random-forest-based RS that used subscriber information, such as age, education, relationship, and occupation, from an insurance company dataset and verified it with an error rate of 0.16. Jain et al. [21] proposed a voting-based RS using airline reviews on the SKYTRAX dataset and verified it with accuracy of 82.7%. Shahbazi et al. [22] proposed an XGBoost-based RS using user-clicked information from an online shopping mall dataset, and verified it with accuracy of 89.6%. Luo et al. [23] proposed a LightGBM-based RS using fashion items, such as color and length, from the Dressipi dataset and verified it with an MRR of 0.206. In contrast to conventional filtering-based RS, AI-based RS can dynamically utilize the data size or features because they use patterns that are not similar between items [35]. Pattern-based RS that use AI have limitations in dynamic situations that change in real time, such as temperature or precipitation. Therefore, conducting research that responds to dynamic situations by reflecting real-time changing external factors and distance information, and subdividing them according to user types, is necessary [36].

## 3. Real-Time Recommendation System for Tourism

**Figure 1:**
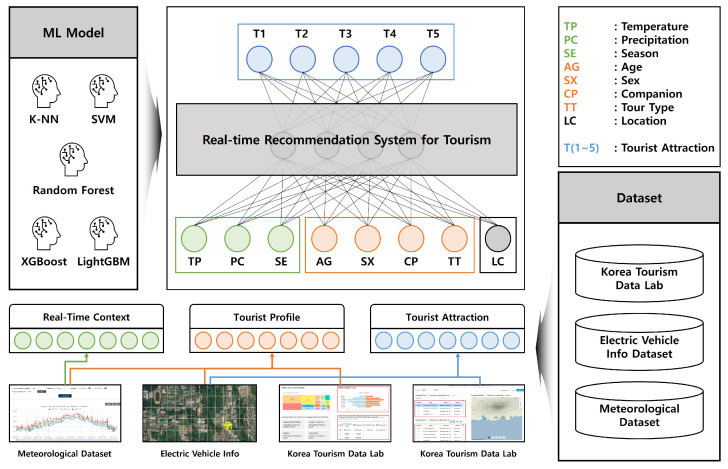
Architecture of real-time recommendation system for tourism.

Handling new data without information is challenging for filtering-based tourist destination RS because of the required data size and the cold start problem. Therefore, research on RS using AI is being conducted [37]. RS using AI can dynamically utilize the size or characteristics of data because they use tourism patterns, ignoring the similarity between items, and achieve remarkable recognition performance with the development of algorithms and computation power [38]. However, RS based on tourism patterns using AI have limitations in dynamic situations because they do not reflect real-time changes in external factors or distance information, such as temperature or precipitation. In this paper, we propose a tourist destination RS using R2Tour to respond to dynamic situations by reflecting real-time changing external factors and distance information and recommending customized tours according to tourist types. As shown in Figure 1, R2Tour recommends the top five nearby tourist destinations by learning a real-time context, including real-time situation information, and tourist profiles, including tourist types, through an ML model. The following section describes the configuration of the tourist attraction corresponding to the dependent variable R2Tour, and the real-time context and tourist profile corresponding to the independent variable.

### 3.1. Tourist Attraction Used for R2Tour

**Figure 2:**
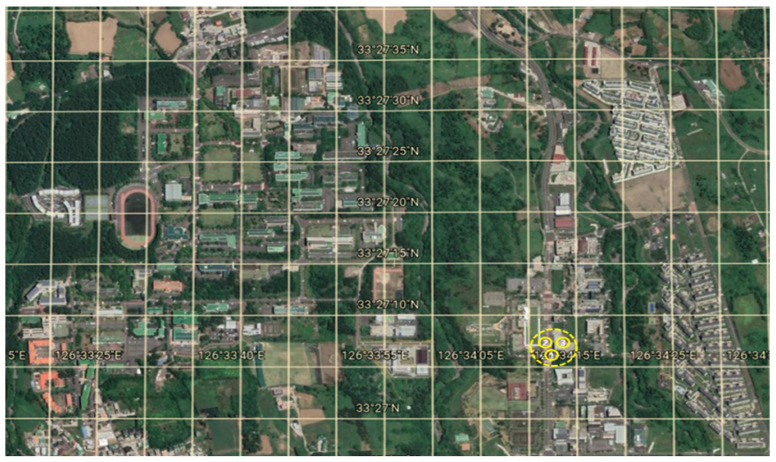
Criteria for EVGPS error range setting.

R2Tour’s independent variable, tourist attraction, consists of information on the central tourist attraction. The top five nearby tourist attractions are obtained according to the current location, which uses the EVGPS and the Korea data lab. The central and related tourist destination information of the Visit Korea data lab consists of information on the main monthly tourist destinations and nearby tourist attractions. The EVGPS measured the movement routes in 1-min units using 52 electric vehicles in the Jeju Smart City pilot city project for three years at KEPCO KDN. The numbers of driving records for each vehicle are listed in Table 2. The central-related tourist attraction information of the Visit Korea data lab used for tourist attractions uses information on the top 10 central tourist attractions and the top five tourist attractions by month, corresponding to Jeju. As shown in Figure 2, the EVGPS sets the error range (50 m) based on the latitude/longitude lines because it is classified as driving owing to positioning errors, even if no actual movement exists. The EVGPS was used in the visiting state if no movement for more than 10 min occurred within the error range. If no movement occurred for more than 200 min, it was excluded from the experiment as a long-term parking state. In addition, the EVGPS classified as visited was used as the visit to the nearest tourist destination within the error range based on the Visit Korea data lab.

### 3.2. Real-Time Context Used for R2Tour

Circumstantial information, such as temperature, precipitation, or season, is a significant factor while visiting tourist destinations, and it changes in real time according to the location [39]. However, existing AI-based RS do not reflect dynamic situations that change in real time because they use tourism patterns. R2Tour learns tourism patterns by reflecting the real-time context and tourism patterns to respond to dynamic situations, addressing the limitations of existing RS. The real-time context, a dependent variable of R2Tour, was extracted from the Korea Meteorological Administration data based on tourist attractions, generated by combining the Visit Korea data lab and EVGPS. The real-time context includes seasonal information on the visit date or measurement date, as well as temperature and precipitation information using Meteorological Agency data.

### 3.3. Tourist Profile Used for R2Tour

R2Tour responds to dynamic situations and uses tourist profiles as a dependent variable to segment customized tour recommendations according to tourist types. Because a tourist profile comprises information on age, sex, companion, and tour type, recommending segmented tourist destinations is possible. Tourist profiles were analyzed based on mobile communication data and keywords on social media. It uses current information for each tourist destination in the Visit Korea data lab. The value corresponding to the highest value based on the data analysis was used as the tourist profile of the destination. The tourist attraction extracted from EVGPS uses similar profile information (error range: −1 to +1) based on the measurement date and time.

## 4. Experimental Results

In this study, we verified the performance of R2Tour using the Jeju tourism dataset in the Python 3.8 environment using an Intel Core i9 processor and an NVIDIA TITAN RTX. The Jeju tourism dataset combines EVGPS, Visit Korea data lab, and Korea Meteorological Administration data and consists of dependent variables for the real-time context and tourist profiles and independent variables for the top five nearby tourist destinations. The real-time context includes weather information such as temperature and precipitation, and time zone information such as season and location. The tourist profiles include gender, age, companion, and tour type. R2Tour applies and evaluates the ML model used in the existing AI-based RS using the real-time context and tourist profiles of the Jeju tourism dataset. R2Tour uses the last year as test data and the rest as learning data to learn past information and predict the future. The following section describes the experimental method, Jeju tourism dataset, and recommendation performance.

### 4.1. Experimental Settings and Jeju Tourism Dataset

R2Tour responds to dynamic situations and compares real-time contexts and tourist profiles by learning several AI models to provide customized tour recommendations according to the tourist type. In this study, K-NN, SVM, random forest, voting, XGBoost, and LightGBM ML models were used in the AI-based RS. The tourist destination RS by R2Tour uses an Intel Core i9 processor and NVIDIA TITAN RTX to learn past information in a Python 3.8 environment and evaluates it by predicting the past year. The Jeju tourism dataset combines EVGPS, Visit Korea data lab, and Korea Meteorological Administration data and consists of dependent variables for the real-time context and tourist profiles and independent variables for the top five nearby tourist destinations. R2Tour’s independent variable, tourist attraction, consists of information on the central tourist attraction, as shown in Section 3.1: the top five nearby tourist attractions according to the current location, which uses the EVGPS and visits the Korea Data Lab. EVGPS only uses ten or more than 200 min of the visit state in the error range (50m), considering the vehicle operating data characteristics. The Visit Korea data lab uses information on the top ten, a monthly tourist destination corresponding to Jeju and Seogwipo, and the top five related to tourist attractions. The real-time context, a dependent variable of R2Tour, is extracted from the Meteorological Agency data based on tourist attractions, as shown in Section 3.2. The real-time context includes seasonal information based on a visit or measurement date and contains temperature and precipitation information using the Meteorological Agency data. The tourist profile, a dependent variable of R2Tour, uses the current status of Visit Korea data lab tourism branches. The tourist profile includes gender, age, companion, and tourism type, as shown in Section 3.3. Tourist attraction extracted from EVGPS uses a tourist profile corresponding to similar values (error range: −1 +1) based on the measurement date and time. The values of each feature are listed in Table 3. To verify the recommendation performance of the model, we utilized Equation (1), which measures the accuracy and the F1-score, which are used when the data are imbalanced. The F1-score evaluation index uses the micro-F1 corresponding to Equation (2) and macro-F1 corresponding to Equation (3) to prove its effectiveness in a data imbalance problem [40].

(1) $$Accuracy=\frac{TP+TN}{TN+TN+FP+FN}$$

(2) $$Micro\;F1\;Score=\frac{2\times T\;P}{2\times T\;P+FP+FN}$$

(3) $$Macro\;F1\;Score=\frac{1}{N}\sum_{i=0}^{N}F1\;Score$$

### 4.2. Recommendation System for Tourism

In the Jeju tourism dataset, 2055 cases (excluding the previous year) were used as the training dataset, and 176 cases corresponding to the latest year were used as the test dataset. Therefore, R2Tour learns past information and is capable of predicting future events. Table 4 lists the results of the evaluation of the RS using R2Tour with the Jeju tourism dataset. When applied to LightGBM, R2Tour was verified with accuracy of 77.3%, micro-F1 of 0.773, and macro-F1 of 0.415. Based on the EVGPS, Jeju tourist destinations were classified into 11 categories, and the results of classifying tourist activity patterns according to weather, season, and time are listed in Table 5. When XGBoost was used, the results of the experiment were verified with accuracy of 80.6%, micro-F1 of 0.806, and macro-F1 of 0.73. The results of the experiment confirmed that real-time situation information, such as weather, season, and time zone information, is a significant factor while visiting tourist destinations. In this paper, the proposed R2Tour trains tourism patterns to recommend tourist destinations according to real-time context and tourist profile information, not the interaction between tourists and tourist destinations. Therefore, even if a tourist has no preference for a new tourist destination, it is possible to recommend it according to tourism patterns such as weather, distance, and tourism type. In addition, unlike existing AI-based RS, it learns tourist destinations according to weather and distance information, so it is possible to respond to changing situational information in real time.

## 5. Conclusions

Recently, there has been a change in the tourism trend towards the Tourism 2.0 paradigm due to the increase in travel experience worldwide and the spread of information acquisition and sharing through the Internet. Tourism 2.0 aims to give new value to the new tourism industry by developing information and technology, providing various experiences and cultures to tourists, and promoting the development of the local economy and environment by pursuing sustainable tourism [41]. FIT is one of the representative tours of the Tourism 2.0 paradigm; travel planning and guidebook applications and tourism recommendation services, which are tourism service tools for FIT in the Tourism 2.0 paradigm, have advantages such as improved customer experiences, time and cost savings, data analysis, and marketing utilization. For tourism service tools, the tourism RS is filtering-based research that recommends tourist destinations according to the relevance of tourists or tourist destinations [42]. Because handling new data without information is challenging for filtering-based RS owing to the required data size and cold start problem, research on RS using AI is being conducted [43]. AI-based RS use tourism patterns, ignoring the similarities between items. It can dynamically utilize the data size or features and achieve remarkable recognition performance by developing algorithms and computation power [44]. However, RS based on tourism patterns using AI have limitations in dynamic situations because they do not reflect real-time changes in external factors or distance information such as temperature or precipitation.

In this study, we proposed a tourist destination RS using R2Tour to respond to dynamic situations by reflecting real-time changing external factors and distance information and recommending customized tours according to tourist types. R2Tour recommends the top five nearby tourist destinations by training an ML model with the real-time context, real-time situation information, and tourist profiles that include tourist types. R2Tour trains tourism patterns to recommend tourist destinations according to real-time context and tourist profile information, not the interaction between tourists and tourist destinations. Therefore, even if a tourist has no preference for a new tourist destination, it is possible to recommend it according to tourism patterns such as weather, distance, and tourism type. In addition, unlike existing AI-based RS, it learns tourist destinations according to weather and distance information, so it is possible to respond to changing situational information in real time. We evaluated R2Tour using the Jeju tourism dataset created by combining EVGPS, Visit Korea data lab, and Korea Meteorological Administration data, and it was verified with accuracy of 77.3%, micro-F1 of 0.773, and macro-F1 of 0.415 in LightGBM. R2Tour responded to dynamic situations and recommended customized tours according to the type of tourist. In addition, classifying tourist activity patterns using XGBoost according to situational information based on EVGPS achieved accuracy of 80.6%, micro-F1 of 0.806, and macro-F1 0.73. The results of the experiment confirmed that real-time situation information, such as weather, season, and time zone information, is a significant factor while visiting tourist destinations. In the future, R2Tour can be expanded to other tasks such as recommending tour schedules [45], travel routes [46], and nearby tourist destinations based on location information. Moreover, R2Tour can be installed in vehicles to recommend nearby tourist destinations or expanded to tasks for the tourism industry, such as a smart target advertising system. The applicability of R2Tour has advantages such as improved customer experiences, time and cost savings, data analysis, and marketing utilization.

## Figures and Tables

**Table 1:** AI-based recommendation system.

Model	Dataset	Performance	Reference
K-NN	Movie Trust	RMSE 0.822	[18]
Support Vector Machine	GTZAN	Accuracy 0.976	[19]
Random Forest	Insurance Companies	Error Rate 0.16	[20]
Voting	SKYTRAX	Accuracy 0.827	[21]
XGBoost	Online Shopping Mall	Accuracy 0.896	[22]
LightGBM	Dresspi	MRR 0.206	[23]

**Table 2:** Central and related tourist attraction information of Visit Korea data lab.

EV_MRID	COUNT	EV_MRID	COUNT	EV_MRID	COUNT	EV_MRID	COUNT
150004	788,894	140032	109,781	140015	39,635	130063	5431
150005	788,893	140017	67,258	140014	39,635	130073	5396
150003	755,789	140046	64,938	150006	39,633	130074	4784
150002	755,789	150009	51,032	140068	21,316	140023	3430
15E005	202,062	150008	51,032	130065	19,057	150001	3084
15E003	202,062	150007	51,032	130062	17,923	140069	1724
15E004	202,062	140027	48,507	130067	17,600	140070	922
15E001	202,062	140028	46,403	130066	17,106	140067	248
15E008	202,062	140013	39,927	130060	16,468	140024	60
15E009	202,062	140012	39,888	130075	11,549	130052	40
15E002	202,062	140019	39,644	130076	9532	140072	32
15E007	202,062	140018	39,635	130077	9157	140020	8
15E006	202,062	140007	39,635	130055	7817	14null	7

**Table 3:** Input and output feature values and details of R2Tour.

Feature	Value	Detail
Temperature	4.8∼29.4	4.8∼29.4 (a monthly average)
Precipitation	9.1∼610.6 (mm)	9.1∼610.6 (a monthly average)
Season	Spring ∼Winter	Spring, Winter, Autumn, Winter
Age	10∼50	10, 20, 30, 40, 50
Sex	Male, Female	Male, Female
Companion	Alone∼Spouse	Alone, Children, Couple, Family, Friend, Parents, Spouse
Tour Type	Cultural Tourism∼Shopping	Cultural Tourism, Mature Tourism, Other Tourism, Shopping
Location	Location	Visit or Plan to Visit Tourist Attractions
Tourist Attraction	Tourist Attraction	Top Five Tourist Attractions Nearby

**Table 4:** R2Tour results for AI-based recommendation system.

MODEL	Result		
	F1-Score (Macro)	F1-Score (Micro)	Accuracy
K-NN	0.092	0.318	0.318
SVM	0.024	0.165	0.165
Random Forest	0.338	0.688	0.688
Voting	0.407	0.761	0.761
XGBoost	0.4	0.75	0.75
LightGBM	0.415	0.773	0.773

**Table 5:** Tourist activity pattern classification results according to real-time context.

MODEL	Result		
	F1-Score (Macro)	F1-Score (Micro)	Accuracy
K-NN	0.714	0.794	0.794
SVM	0.298	0.52	0.52
Random Forest	0.695	0.778	0.778
Voting	0.282	0.514	0.514
XGBoost	0.731	0.806	0.806
LightGBM	0.583	0.71	0.71

## Data Availability

Restrictions apply to the availability of these data. Data was obtained from Visit Korea Data Lab and are available https://datalab.visitkorea.or.kr/ with the permission of Visit Korea Data Lab.

## Abbreviations

**FIT**	Free/Foreign Independent
**CF**	Collaborative Filtering
**CB**	Content Filtering
**RS**	Recommendation System
**AI**	Artificial Intelligence
**R2Tour**	Real-Time Recommendation System for Tourism
**ML**	Machine Learning
**EVGPS**	Electric Vehicle Information
**KEPCO KDN**	Korea Electric Power Corporation Knowledge Data Network
